# Role of dynamic contrast-enhanced magnetic resonance imaging in the diagnosis and management of vascular lesions of the head and neck

**DOI:** 10.17305/bjbms.2021.6019

**Published:** 2021-08-19

**Authors:** Raluca Petea-Balea, Manuela Lenghel, Horatiu Rotar, Cristian Dinu, Simion Bran, Onisor Florin, Rares Roman, Simona Senila, Csaba Csutak, Ciurea Anca

**Affiliations:** 1 Department of Radiology and Medical Imaging, County Clinical Emergency Hospital, Cluj-Napoca, Romania; 2 Department of Radiology, Faculty of Medicine, “Iuliu Hațieganu” University of Medicine and Pharmacy, Cluj-Napoca, Romania; 3 Department of Cranio-Maxillofacial Surgery, “Iuliu Haţieganu” University of Medicine and Pharmacy, Cluj-Napoca, Romania; 4 Department of Maxillofacial Surgery and Implantology, Faculty of Dentistry, “Iuliu Hațieganu” University of Medicine and Pharmacy, Cluj-Napoca, Romania; 5 Department of Cranio-Maxillofacial Surgery and Radiology, Faculty of Dental Medicine, “Iuliu Hațieganu” University Of Medicine and Pharmacy Cluj-Napoca, Romania; 6 Department of Dermatology, “Iuliu Haţieganu” University of Medicine and Pharmacy, Cluj-Napoca, Romania

**Keywords:** Vascular malformation, dynamic contrast-enhanced magnetic resonance, head and neck, hemangioma, post-therapeutic evaluation

## Abstract

Vascular anomalies comprise a wide and heterogeneous group of lesions that may be found in all parts of the body, with most of the cases of vascular malformations involving the head-and-neck region. Ultrasound (US) is the reliable first-line imaging technique to assess flow parameters. However, in some cases, US fails to depict the real extent of the lesions. On the other hand, magnetic resonance imaging (MRI) allows the evaluation of the full extension and anatomic relationship of the vascular anomalies with the neighboring structures and provides hemodynamic characterization using dynamic contrast-enhanced MRI (DCE-MRI), avoiding unnecessary invasive catheter-based procedures. DCE-MRI angiography can make a distinction between low- and high-flow vascular anomalies and it is useful for selecting adequate therapy and appreciating prognosis. The aim of this paper is to review the role of DCE-MRI in the evaluation of flow characteristics and lesion extent in vascular anomalies of the head-and-neck region.

## INTRODUCTION

Vascular anomalies comprise a large spectrum of lesions that involve mainly the head-and-neck region and often associate significant morbidity. For a long time, vascular anomalies have been misclassified, generating considerable confusion among the clinicians and antagonistic results in terms of treatment strategies and patient outcome [1]. Multiple classification systems have been proposed for vascular anomalies, but a true milestone was achieved in 1982 by Mulliken and Glowacki and it was based on histological components. The proposed system divided vascular anomalies into two major categories: Vascular malformations (MAV) and hemangiomas [2]. In 1996, the International Society for the Study of Vascular Anomalies (ISSVA) adopted and upgraded the previous existing classification systems differentiating vascular MAV from vascular tumors, represented especially by hemangiomas [3].

Although clinical history and findings of the physical examination may be sufficient to indicate the diagnosis in the initial phase, additional imaging tools are mandatory for the proper characterization of the head-and-neck lesions due to their unique implications [4]. Imaging assessment of the vascular lesions is based on various imaging techniques, such as ultrasound (US), contrast-enhanced computed tomography (CT), magnetic resonance imaging (MRI), and digital subtraction angiography (DSA) [5].

Due to the complexity of the anatomy of the head-and-neck region, the extent of the lesions and their topographic relationship to the neighboring tissues are best assessed using MRI. Besides morphological features of the lesions, MRI complemented by dynamic contrast-enhanced MRI (DCE-MRI) sequences is used to characterize flow dynamics and can differentiate between high-flow and low-flow vascular MAV, without using unnecessary invasive catheter-based arteriography.

In this way, MRI has become the most valuable imaging modality in the analysis of vascular MAV and hemangiomas in many specialized health-care units [4,6]. The role of the US in the assessment of vascular lesions is well established and it is often the first step of the diagnostic process. US with color Doppler mode allows anatomical localization of the lesion, supplies information about the distribution and density of vascularization of the lesion and, like MRI, it can make a differentiation between high-flow and low-flow MAV and also between hemangiomas and vascular MAV. Nonetheless, the US is not a perfect tool in diagnostic imaging because it fails to depict the real extent of the lesions due to the limited field of view and inappropriate penetration of deeper structures [7-9].

CT angiography can be performed to assess the vascular architecture of the lesions, patterns of enhancement, presence of calcification, and phleboliths and to determine involvement of surrounding structures. It has limited value in the pediatric population due to the use of ionizing radiation, being used only to assess bone involvement [5].

Conventionally, conventional angiography has been considered the “gold standard” for diagnosing vascular lesions. Nowadays, catheter-based procedures are used for diagnostic purposes only in indeterminate cases of high-flow MAV after all other non-invasive methods have been exhausted. For high-flow lesions, transarterial embolization remains the main treatment option [6]. The purpose of this article is to review the lesion extent and hemodynamic characteristics of the vascular anomalies of the head and neck using conventional MRI and DCE-MRI.

## CLASSIFICATION SYSTEM AND NOMENCLATURE OF VASCULAR ANOMALIES

Under the umbrella of vascular lesions, there is a broad spectrum of disorders ranging from simple, unremarkable lesions to dangerous, even life-threatening lesions which can be either self-isolated or associated with complications [10].

The earliest attempt to classify the vascular anomalies was proposed in the mid-18^th^ century by Virchow and was based on histological features [11]. Since then, the classification system has suffered multiple and various changes that improved the knowledge of vascular lesions and helped clinicians in the process of finding better treatment solutions. In 1982, Mulliken and Glowacki described the first significant classification system, which divided vascular lesions into vascular MAV and hemangiomas [2].

In 1993, Jackson et al. elaborated a new system based on the one proposed by Mulliken and Glowacki and stratified vascular MAV according to their flow velocity parameters as low-flow and high-flow MAV [12]. At the first workshop of ISSVA in 1996, these systems were integrated and developed to achieve a uniform classification. Vascular anomalies were further divided into proliferative vascular lesions (tumors) and vascular MAV. High-flow MAV are represented by arteriovenous (AV) MAV and AV fistulas (AVFs). Depending on the predominant type of vascular channel identified, low-flow MAV can be capillary, venous, and lymphatic. The association of one or more vascular MAV may occur, resulting in mixed MAV such as capillary venous, lymphatic venous MAV or other combinations [3]. Based on recently added information in the field of biology and genetics, the ISSVA Classification of Vascular Anomalies was upgraded at the last workshop in Melbourne, Australia (April 2014) and revised in May 2018. The updated (2018) classification includes links to certain related clinical parameters, such as coagulation abnormalities as well as genetic syndromes that might be associated [10,13].

Interestingly, the nomenclature system and classification remain a challenge despite the long-standing efforts, therefore implementation in everyday practice might take a while. The most common mistake refers to the inappropriate use of the term “hemangioma,” which is often attributed to designate both different types of tumors and vascular MAV [2].

## CLINICAL PRESENTATION

### Vascular tumors

Hemangiomas are the most common vascular tumors of children, involving the head-and-neck region in more than half of the cases (60%) [8,14]. These benign tumors are represented mostly by infant hemangioma and congenital hemangioma and less commonly by several rare entities such as kaposiform hemangioendothelioma, tufted angioma, pyogenic granuloma, and angiosarcoma. Hemangioma of infancy or infantile hemangioma tends to affect the newborn, with a higher incidence among premature infants (12-23%) [8,14]. They can be present or non-apparent at birth and are defined by a growth phase followed by an involution phase, with slow and almost complete regression by the age of 5 years. Superficially located hemangiomas, also known as “strawberry hemangiomas,” appear as red masses with clear borders. Treatment is required only in case of complications or for esthetic consideration in case of facial deformity. Congenital hemangiomas, on the other hand, are invariably present at birth and can follow three evolution directions: Non-involution, partial involution, or rapid involution [13,14].

Usually, they present as a single lesion, but can sometimes be multiple, diffuse, or can associate with other conditions resulting in different syndromes. Kaposiform hemangioendothelioma belongs to borderline vascular tumors with intermediate aggressiveness and metastatic potential. Unlike previously mentioned hemangiomas, this condition can be part of Kasabach-Merritt syndrome, characterized by pediatric hemangiomas associated with consumption coagulopathy [15].

### Vascular MAV

Vascular MAV are present at birth, although not always noticeable. Unlike hemangiomas, they show no regression in time and tent to increase in size as a consequence of hormonal changes during puberty or pregnancy, predisposing to complications. They can be divided, based on the type of the flow, into low-flow and high-flow vascular MAV [7,16].

### Low-flow MAV

Almost 40% of the venous MAV are found in the head-and-neck region. On clinical examination, they often tend to present as soft, compressible, non-pulsatile subcutaneous masses located especially around the mouth, lips, tongue, cheek, scalp, and neck. Blue discoloration of the skin or mucosa is seen when the lesions extend to superficial structures. As a result of stagnant blood flow through the abnormal vascular channels, thrombosis may occur, leading to phleboliths formation [7,17,18].

**FIGURE 1:**
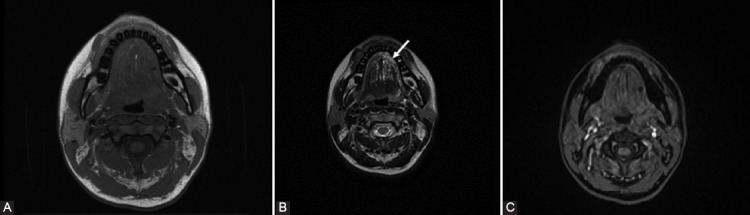
A 21-year-old woman with previous surgery for microcystic lymphatic malformation and local recurrence at the level of the left hemi-tongue extended to vallecula. (A) Axial T1WI depicts multiple small tubular structures, isointense, relative to the muscles. (B) On T2WI, the structures appear hyperintense (arrow). (C) Axial gadolinium -enhanced fat-suppressed T1WI shows absence of enhancement of the dilated lymphatic vessels.

Lymphatic MAV are caused by the abnormal development of the lymphatic system with formation of multiple cystic structures that fail to communicate with peripheral draining vessels. Most of the lesions are located in the head and neck (70-80%) with predilection for posterior triangle and submandibular spaces (Figure 1). Based on morphological features, MAV are divided into macrocystic, when cysts are larger than 2 cm in diameter, microcystic, when they are composed of cysts smaller than 2 cm, and mixed lesions. Clinically, they present as soft-tissue structures that are non-compressible and can cause various symptoms depending on location and size. Infection is a frequent complication leading to enlargement of the lesion, which becomes painful and firm [5,17,19].

Capillary MAV also known as “port wine stain” consist of a red discoloration of the skin that is present in 0.3% of children. In general, the dilated capillary bed is limited to the dermis and mucosa, but sometimes it can be part of Sturge-Weber or Klippel-Trenaunay syndrome. Sturge-Weber syndrome is a phakomatosis which joins capillary MAV following the territory of trigeminal nerve and pial angiomas [4,15].

### High-flow MAV

High-flow MAV of the head-and-neck region are congenital vascular anomalies which are present at birth and routinely associate skin discoloration, local warmth, and presence of an underlying pulsatile bruit. When AVMs are located in the face region, they can be aggressive and lead to considerable facial deformity and bone destruction. They can give rise to serious complications secondary to thrombosis, infection, and traumatic events such as ischemic events, ulceration, and hemorrhage that may endanger the patient’s life. High cardiac output failure is rarely seen as an initial presentation [7,20,21].

### Imaging of vascular anomalies

Imaging of vascular anomalies is imperative and has two major purposes: First, to establish the accurate extent and site of the vascular lesion, and second, to provide information about hemodynamic parameters. It is of paramount significance to discriminate between low-flow and high-flow lesions, because this knowledge will strongly impact the planning of further treatment.

Several imaging modalities (color Doppler US, CT angiography, MRI/DCE-MRI, and DSA) are used for the assessment and characterization of vascular lesions, each with its advantages and limitations. Since the pediatric and adolescent population are more prone to present vascular lesions, the use of ionizing radiation should be limited as much as possible [5,8].

US is often the first step of diagnostic procedure allowing assessment of the size, morphology, and vascular flow of the lesions without using radiation. Like MRI, it is a non-invasive imaging modality that can make a clear distinction between low-flow and high-flow vascular lesions and is used in the diagnosis and follow-up of the patients. Small, superficial lesions are best assessed using US [9].

CT can also provide valuable information in long-standing lesions and lesions involving bony structures, but is reserved for adolescents and adult patients. Calcification and phleboliths are best detected on non-contrast CT [8,21]. Due to the high risk of vascular injury and exposure to ionizing radiation, DSA tends to be no longer the “gold standard” method for vascular lesions characterization. It still plays an important role in the indeterminate cases of high-flow MAV [5,22].

The morphology and extent of head-and-neck vascular lesions and their topographic relationship to surrounding structures can be easily depicted by MR imaging due to its high spatial resolution and multiplanar imaging. In recent years, functional MRI imaging techniques such as DCE-MRI have remarkably contributed to the diagnosis of vascular lesions of the head-and-neck region. DCE-MRI is an effective method in differentiating between low-flow and high-flow vascular lesions, improving diagnostic accuracy, and replacing almost entirely invasive DSA in the diagnosis and follow-up of the vascular anomalies [21,23,24].

### MRI protocol

For the evaluation of the head-and-neck vascular lesions, small surface coils are used in general, but with sufficient length to cover the area of interest. In case of palpable lesions, a skin marker can be placed to make sure the whole lesion is included within the field of view [7,17].

The MRI study protocol consists of standard sequences including multiplanar spin echo (SE) or fast SE T1-weighted imaging (T1WI) for basic anatomic evaluation, and SE T2-weighted imaging (T2WI) with or without fat saturation images for the lesions extent and their relationships with nearby anatomic elements such as skin, subcutaneous tissues, muscle, bones, and neurovascular structures. Fast short-tau inversion recovery (STIR) sequences can be also used, but they are more susceptible to motion artifacts. Gradient recalled echo (GRE) T2*WI can depict increased signal within high-flow vessels and hemosiderin deposits [6,7,21].

Assessment of flow within the lesion can be made currently with DCE-MRI using time-resolved sequences, such as time-resolved imaging of contrast kinetics (TRICKS) or time-resolved angiography with interleaved stochastic trajectories (TWIST) allowing not only great space resolution but also high temporal resolution (2-4 s), mandatory in distinguishing between high-flow and low-flow lesions [6,25,26].

In 2002, van Rijswijk et al. [27] in a study performed on subjects with clinical suspicion of peripheral vascular MAV, found that adding DCE-MRI sequences to conventional MRI sequences can increase specificity of MRI from 24–33% to 95%. Furthermore, in a prospective study by Lidsky et al. [6], DCE-MRI proved to be a successful tool to diagnose abnormal flow parameters in at least 83.8% of cases.

Distinction between arterial and venous phase enhancement is often based on the visual assessment of the dynamic sequences. The previous studies establish a cutoff value of 6 s to differentiate arterial phase from venous phase. Thereby, early enhancement reflecting arterial phase is referred to be <6 s, while late enhancement reflects the venous phase >6 s and is evocative for pure venous MAV [25].

A study conducted by Higgins et al. [25] in 2015 showed that performing TWIST sequences after administration of gadofosveset trisodium provide early assessment of hemodynamic parameters leading to precise classification. In addition, utilization of gadofosveset trisodium allows the reduction of the regular contrast medium dosage by two-thirds.

In a study conducted by Hassanien et al. in 2017, TRICKS-MRA proved to be a precise tool in the determination of internal vascular architecture of the facial vascular anomalies, specifying feeding arteries and drainage veins without using invasive procedures, and making the therapeutic approach easier [22].

### MRI imaging features of vascular tumors

**FIGURE 2:**
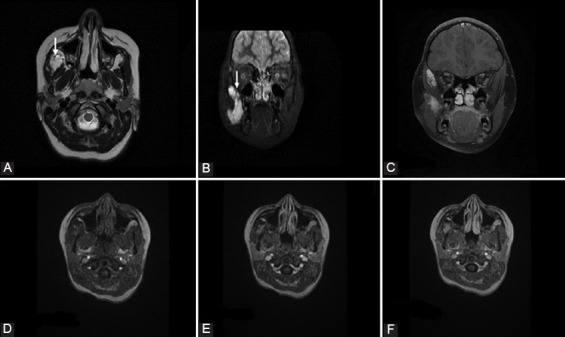
A 9-year-old girl with progressive swelling of the left side of the cheek diagnosed with congenital hemangioma. (A) Axial T2W1 shows a focal, well-delineated lesion with hyperintense signal at the level of right masticator space. (B and C) Short-tau inversion recovery images show cranial extension of the lesion. Signal voids in the lesion (arrows) represent high-flow vessels. No perilesional edema was identified. Axial gadolinium-enhanced fat-suppressed T1WI after 5 s (D), 30 s (E), and 70 s (F) shows increased progressive enhancement, findings consistent with congenital hemangioma.

**FIGURE 3:**
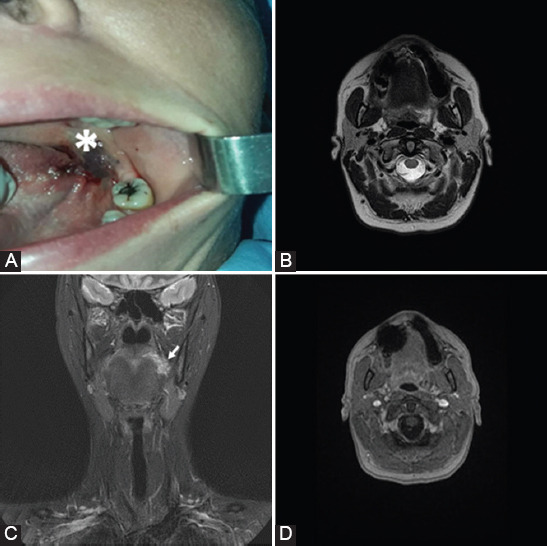
A 41-year-old woman with swallowing difficulty diagnosed with capillary hemangioma. (A) Photograph shows a soft dark red lesion behind the second molar on the left (*). (B) Axial and (C) coronal T2WI evidence a focal lesion with high-intensity signal at the level of left sulcus terminalis of the tongue. (D) Axial gadolinium-enhanced fat-suppressed T1WI shows absence of enhancement at the level of the lesion.

Infantile hemangiomas can exhibit various aspects on MRI according to the biological phase. Therefore, they appear as well-defined, lobulated lesions with intermediate signal intensity on T1WI and high signal intensity on T2WI. When high-flow feeding arteries and draining veins are present, flow voids can validate on SE images (Figures 2 and 3). DCE-MRI will demonstrate early and homogeneous enhancement of the lesion. Throughout the involution stage, the lesion tends to be replaced by fatty tissue giving a heterogeneous appearance with spots of increased signal intensity on T1W1. Furthermore, enhancement of the lesion is less strong in the involution stage. MRI features of congenital hemangiomas resemble the ones of infantile hemangioma [7,14,21,28].

In comparison with congenital and infantile hemangioma, kaposiform hemangioendothelioma tends to have indistinct borders, smaller feeding and draining vessels, and infiltrating growing pattern with destruction of the tissue involved [7,17,23].

### MRI imaging features of vascular MAV

**FIGURE 4:**
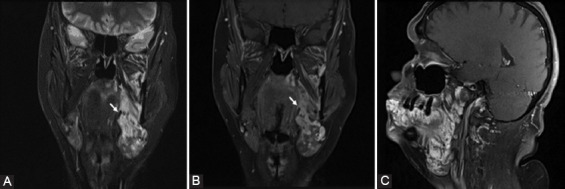
A 51-year-old woman with a lump in the right submandibular region confirmed by magnetic resonance imaging to be a venous malformation. (A) Coronal short-tau inversion recovery image demonstrates the presence of heterogeneous lesions, predominantly showing high-intensity signal at the level of the entire hemi-face on the left, involving superficial and profound tissues as well. (B) Coronal and (C) sagittal gadolinium-enhanced fat-suppressed T1WI demonstrates the presence of multiple serpiginous vessels. Phleboliths may be observed (arrow).

In MRI examination, venous MAV often present as septate, ill-defined lesions with intermediate to low signal intensity on T1W1. On T2WI and STIR images, lesions present high-intensity signals corresponding to dilated venous structures (Figure 4). The presence of the calcified thrombi known as phleboliths represents the hallmark of the venous MAV and produces flow voids on both T1WI and T2WI and high signal on GRE T2* [7,21]. High-protein content and hemorrhage demonstrate high signal intensity on T1WI. Pure VMs show delayed enhancement after contrast administration. By contrast, mixed capillary venous MAV exhibit earlier enhancement [8,23].

Lymphatic MAV appear as multiloculated cystic structures with decreased or intermediate signal on TIWI and high signal on T2WI. Fluid-fluid levels are not unusual and can be seen especially in cases of previous internal hemorrhage or debris from infection. Due to the cystic components enhancement is absent, although peripheral enhancement corresponding to vessels within the septa may be remarked. In microcystic types, the cyst may not have clearly recognizable cystic spaces and the lesion may present with moderate increased signal after gadolinium-based contrast material injection due to septal enhancement [7,28,29].

Although pure capillary MAV show only mild skin thickening on MRI, imaging assessment is necessary because they can be only the “tip of the iceberg” of a more complex malformation (Figure 4) [21].

AVMs are characterized by the presence of a nidus, which is the convergence of multiple high-flow serpentine abnormal feeding arteries and draining veins, creating a direct communication between the arterial and venous systems. These MAV lack the normal capillary network between vessels. Central nidus can sometimes present calcifications [7,21,30]. AVF, on the other hand, is defined by a single communication between a venous and an arterial structure [21].

**FIGURE 5:**
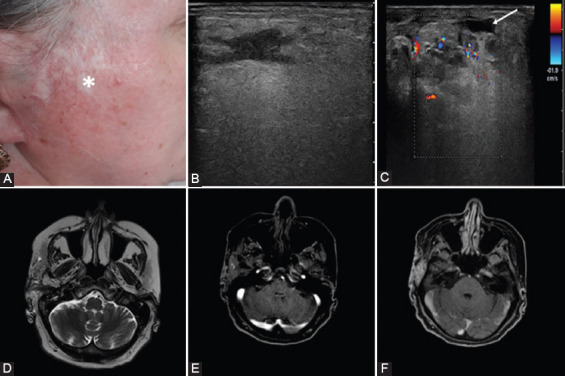
A 52-year-old woman with painful swelling at the level of the right parotid diagnosed with high-flow vascular malformation. (A) At clinical examination, atrophic changes of the skin were noted (*). (B) Transverse gray-scale sonogram shows an irregular hypoechoic lesion that extends into surrounding soft tissues which present edema. (C) On color Doppler sonogram, the lesion is composed of multiple abnormal vessels. Notice the presence of a superficial thrombosed vein (arrow). (D) Axial T2W1 shows a lobulated soft-tissue lesion with heterogeneous signal on T2WI. (E) Axial gadolinium-enhanced fat-suppressed T1WI demonstrates the presence of multiple vessels, some with early enhancement (5 s after contrast material injection) – arteries with origin in the right external carotid artery and (F) some vessels with late enhancement (70 s after contrast material injection).

Defining features of high-flow MAV include tortuous signal voids, minimal or insignificant parenchymal tissue, and intraosseous extension of the lesion seen as low signal marrow intensity on T1WI. Hemorrhage and intravascular thrombosis can lead to high signal areas on T1WI [7]. On DCE-MRI, high-flow MAV are identified by the presence of the flow within the lesion at the moment or before the visualization of the arterial flow within neighboring normal vessels (Figure 5). Early and intense venous enhancement is typically seen in AVMs [6].

### Management of vascular anomalies

A multidisciplinary team approach is required to integrate surgical and non-surgical interventions for optimum results, as these lesions are laborious to treat and recur often [5,31]. There is a wide arsenal of treatment options that can be used for treating hemangiomas depending on the different stages of growth. Proliferative hemangiomas benefit from drug therapy followed by laser therapy and sclerotherapy. Clinical observation should be restrained only for hemangiomas which are without visible growth or in the regression phase [32].

Only symptomatic low-flow MAV are suitable for treatment. Options include compression garments and transcutaneous sclerotherapy completed by surgery when necessary [5]. High-flow MAV are most often treated with transarterial catheter-based techniques which produce obliteration of the middle part of the lesion, completed by surgical resection. Esthetic reconstruction with flaps can stop their recurrence [21,30,33].

### Post-therapeutic appearances

The follow-up management of patients with vascular lesions should be adapted to the particularities of each patient and varies depending on the type of malformation, characteristics and affected region, as well as the type of the treatment. MRI is the reference imaging modality for post-therapeutic evaluation of the lesions [21].

Effectiveness of sclerotherapy in patients with venous MAV is usually assessed after 3 months after therapy. Immediate after the procedure, a strong inflammatory reaction and thrombosis of the malformation leads to an increased signal on T2WI. MRI angiography reveals absence of enhancement in the central part of the treated lesion with intense rim enhancement due to reactive hyperemia. After 3 months, peripheral enhancement is no longer seen and the central scar appears dark on T1WI and STIR images.

High-flow MAV benefit from transarterial embolization which leads to thrombosis of the lesion and eradicates the nidus of the lesion. DCE-MRI may depict reduced or absent shunting and absence of the contrast in venous vessels in early phase. Prompt evaluation is mandatory for the early recognition of residual MAV and planning the next stages of treatment [7,21,34,35].

## CONCLUSION

Vascular anomalies of the head and neck are complex lesions that are often present at birth and have variable growth patterns, leading to esthetic issues and sometimes life-threatening complications. Although the clinical examination and history of patient are important, MRI with DCE-MRI sequences proved to be the cornerstone imaging modality for the proper characterization of vascular lesions of the head-and-neck region before treatment, by classifying them into high-flow vascular lesions and low-flow vascular lesions. Furthermore, MRI is an excellent tool for the evaluation of treatment outcome and the best method to be chosen in case of complex MAV occurring in children and adolescents.
